# Effectiveness of Kinesio Taping Combined With Neuromuscular Control Exercises on Pain and Function in Knee Osteoarthritis: A Multicenter Assessor‐Blinded Randomized Controlled Trial Protocol

**DOI:** 10.1002/hsr2.73125

**Published:** 2026-09-07

**Authors:** Md. Zahid Hasan, Kazi Md Azman Hossain, Mohammad Anwar Hossain, Md Obaidul Haque, Farjana Sharmin, Farzana Sharmin, Md. Zahid Hossain

**Affiliations:** ^1^ Centre for the Rehabilitation of the Paralysed (CRP) Dhaka Bangladesh; ^2^ Department of Physiotherapy and Rehabilitation Jashore University of Science and Technology (JUST) Jashore Bangladesh; ^3^ Department of Physiotherapy Bangladesh Health Professions Institute (BHPI) Dhaka Bangladesh

**Keywords:** kinesio taping, knee osteoarthritis, neuromuscular control exercise

## Abstract

**Background and Aims:**

Knee osteoarthritis (KOA) is a leading cause of functional impairment and decreased quality of life. Exercise is the recommended approach; however, adjunct interventions such as kinesio taping (KT) have gained attention for potential to reduce pain, improve proprioception, and enhance functional performance. Despite its growing clinical use, research on the effectiveness of combining KT with neuromuscular control exercises (NCE) in KOA remains limited. Therefore, this study aimed to assess the effectiveness of KT combined with NCE in reducing pain and improving functional outcomes in KOA.

**Methods:**

This randomized controlled trial is recruiting 80 participants with KOA. Participants are randomly allocated (1:1) to either an experimental group receiving KT combined with NCE or a control group receiving sham KT with NCE. Both groups are undergoing supervised intervention sessions three times per week for 8 weeks. Primary outcomes include pain intensity (Visual Analog Scale, VAS), pressure pain threshold (PPT—digital pressure algometer), and functional status (Western Ontario and McMaster Universities Osteoarthritis Index, WOMAC). Secondary outcomes include lower limb muscle strength (modified sphygmomanometer), knee range of motion (goniometer), and functional mobility (Timed Up and Go test, TUG). Outcomes are planned to be assessed at baseline, post‐intervention (8 weeks), and follow‐up (6 months). Repeated‐measures ANOVA will assess group, time, and group × time interaction effects, with between‐group differences and 95% CIs reported according to the intention‐to‐treat principle.

**Results:**

The trial is expected to generate evidence on whether adding KT to NCE yields greater improvements in pain modulation, physical function, and mobility in KOA.

**Conclusions:**

This study will address a crucial evidence gap in multimodal, non‐pharmacological rehabilitation for KOA. Findings could support evidence‐based clinical decisions and provide a cost‐effective addition to exercise therapy, especially in resource‐limited settings like Bangladesh. Results might also guide future multicenter trials and standardized rehabilitation protocols for KOA.

**Trial Registration:**

CTRI/2025/08/093317 [Registered on: 20/08/2025]; trial registered prospectively.

**Abbreviations**
BMI: Body Mass Index
CCI: Charlson Comorbidity Index
CONSORT: Consolidated Standards of Reporting Trials
ICC: Intraclass Correlation Coefficient
KOA: Knee Osteoarthritis
KT: Kinesio Taping
NCE: Neuromuscular Control Exercise
RCT: Randomized Controlled Trial
ROM: Range of Motion
SPIRIT: Standard Protocol Items: Recommendations for Interventional Trials
SPSS: Statistical Package for the Social Sciences
TUG: Timed Up and Go Test
VAS: Visual Analog Scale
WOMAC: Western Ontario and McMaster Universities Osteoarthritis Index

## 1 Introduction

Knee osteoarthritis (KOA) is one of the most prevalent forms of osteoarthritis, affecting approximately 365 million individuals worldwide [1]. It is characterized by synovitis, cartilage degeneration, and the formation of osteophytes, involving both contractile (muscle) and non‐contractile (cartilage, bone, ligament, tendon, synovium, meniscus) structures [2, 3]. KOA has become a significant cause of impairment among the elderly [4]. As the global elderly population continues to grow, an increasing number of individuals suffering from KOA are experiencing pain, oedema, stiffness, and functional impairments. Current studies estimate that around 18% of elderly men and 27% of elderly women suffer from KOA. Furthermore, these figures are anticipated to increase in the upcoming decades [5]. This condition impairs joint and muscle function, causing balance and gait issues [6]. Therefore, KOA is less avoidable than commonly believed.

Current KOA treatments aim to alleviate relevant symptoms through various methods, such as pharmacological, non‐pharmacological, and surgical interventions [4]. Non‐pharmacological options include manual therapy, physiotherapy modalities, taping techniques, and therapeutic exercises, all of which are utilized to treat KOA [7]. Among these, the utilization of kinesio taping (KT) has gained increasing prevalence among patients with KOA. Dr. Kenzo Kase pioneered the development of the KT treatment in Japan during the 1970s [4]. KT has the potential to enhance muscular strength and improve knee condition. KT is an elastic adhesive tape applied to the skin. It is proposed that pain and function be influenced through altered cutaneous afferent input, proprioceptive modulation, neuromuscular facilitation, and potential effects on local circulation and lymphatic flow. Patients with KOA may benefit from KT, as it can alleviate pain, reduce swelling, improve ligament function, increase range of motion (ROM), and stabilize the knee joint [8, 9]. KT is gaining recognition as a physical treatment for KOA, with more clinicians and therapists adopting it [9]. However, evidence regarding KT's mechanism and effectiveness remains hypothetical, with mixed evidence reported in the literature. Although some high‐quality research has been conducted on KT in KOA patients, the overall conclusion about its impact remains inconclusive [5]. A 2013 systematic review states that KT interventions are not recommended for these musculoskeletal conditions [10]. Conversely, three meta‐analyses have demonstrated that KT can reduce pain and improve knee function compared to sham KT [11, 12, 13]. Another study in 2020 highlighted the significant impact of KT compared with physiotherapy on pain and functional improvement [14]. Additionally, recent research indicates that KT, or KT combined with therapeutic exercise, can have a substantial positive effect on patients with KOA [15]. Appropriate therapeutic exercise prescriptions generally benefit KOA patients, with neuromuscular control exercises (NCEs) being particularly effective. NCE has the potential to improve balance, muscle activation, functional alignment, and joint stability in individuals with KOA [16]. A study shows NCE improves cartilage and lowers knee loads in mild KOA patients [17]. Personalized, advanced NCE improves patient outcomes and physical abilities, such as movement independence and knee strength, even in older, severe KOA patients [18]. Previous research suggests NCE may be more effective than pharmacotherapy in alleviating long‐term symptoms like swelling and stiffness, addressing mechanical issues, and reducing side effects from analgesics and anti‐inflammatory drugs [16]. A prior study examined the impact of an NCE program utilizing non‐elastic taping on the knee joint in patients with KOA [18]. However, no research has yet measured the effectiveness of combining KT and NCE. Nonetheless, this combination may represent an effective treatment strategy for managing KOA. Based on this, we have planned this study.

Therefore, the primary objective of this randomized controlled trial (RCT) is to evaluate the impact of 8 weeks of KT combined with NCE on pain reduction and functional improvement in patients suffering from KOA. Additionally, we intend to examine the impact of KT and NCE on other outcome measures, such as muscle strength, ROM, and functional mobility. We also aimed to assess the potential long‐term advantages of integrating KT with NCE beyond the immediate post‐intervention period, through a 6‐month follow‐up, and to assess the safety of this intervention. We hypothesize that participants receiving KT in addition to NCE will demonstrate significantly greater improvements in pain, physical function, and neuromuscular performance than those receiving sham KT with NCE.

### 1.1 Research Question

Among adults aged 45–70 years with radiographically confirmed KOA, does KT combined with an NCE program, compared with sham KT combined with the same exercise program, lead to greater improvements in pain, physical function, and relevant outcomes immediately after the 8‐week intervention and at 6‐month follow‐up?

## 2 Methods

### 2.1 Study Design

**Figure 1:**
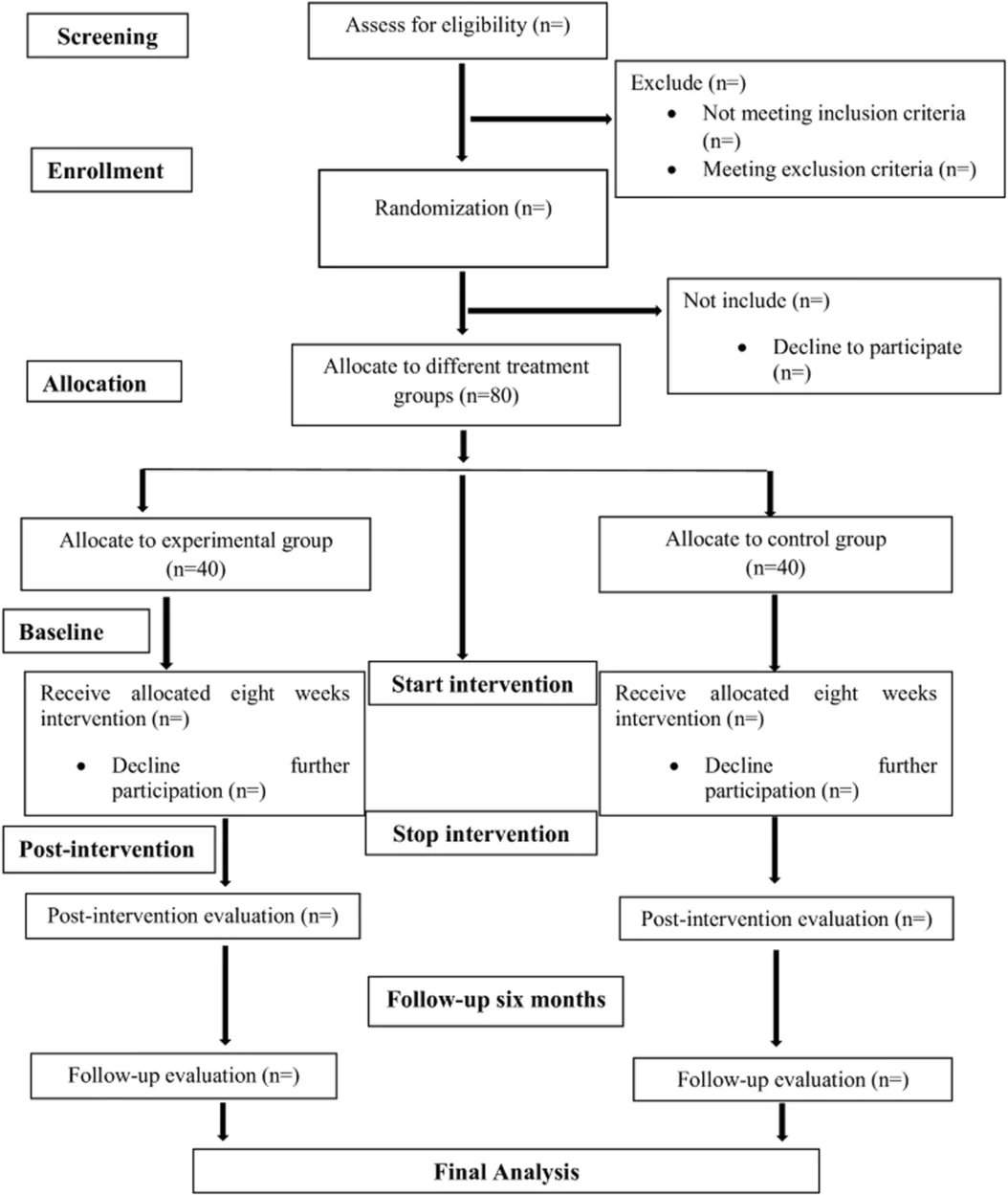
CONSORT flow diagram.

This study is a multicenter, assessor‐blinded, RCT comparing the effects of KT with NCE (experimental group) versus sham KT with NCE (control group) in a 1:1 ratio among 80 KOA patients in Bangladesh. Each group's intervention lasts 8 weeks, with three sessions per week, followed by a 6‐month follow‐up. Primary and secondary outcomes were planned to be measured at baseline, post‐intervention, and follow‐up. The study obtained ethical approval and was registered in the Clinical Trial Registry. To ensure transparency, the study adhered to the 2013 SPIRIT (Standard Protocol Items: Recommendations for Interventional Trials) guidelines (Table 1). Additionally, the CONSORT (Consolidated Standards of Reporting Trials) statement guidelines will be utilized to ensure transparent reporting of the findings (Figure 1).

**Table 1:** SPIRIT guidelines.

Timepoint	Enrollment	Allocation		Post‐allocation	
	−T_1_	T_0_	T_1_	T_2_	T_3_
Enrollment					
Eligibility screening	X				
Informed consent		X			
Demographic and clinical assessment			X		
Group allocation		X			
Intervention					
Experimental group			X	X	
Control group			X	X	
Assessment					
VAS			X	X	X
PPT			X	X	X
WOMAC			X	X	X
Muscle strength			X	X	X
ROM			X	X	X
TUG			X	X	X

*Note:* −T_1_ = pre‐study enrollment; T_0_ = group allocation; T_1_ = baseline before intervention; T_2_ = measurement after 8 weeks; T_3_ = measurement after 6 months.

Abbreviations: PPT, pressure pain threshold; ROM, range of motion; TUG, Timed UP and Go test; VAS, visual analog scale; WOMAC, Western Ontario and McMaster University Osteoarthritis Index.

### 2.2 Participants and Recruitment

Participants meeting the eligibility criteria were recruited from the four participating branches of the Centre for the Rehabilitation of the Paralysed (CRP), Bangladesh, through routine physiotherapy services, clinical records, and referrals. A trained research assistant who was not involved in intervention delivery or outcome assessment conducted the initial screening using a standardized eligibility checklist. Recruitment commenced in September 2025 and is expected to continue through December 2026, including follow‐up. Eligible individuals received detailed information about the study procedures, potential benefits and risks, confidentiality, and their right to withdraw at any time without affecting their usual care. Written informed consent was obtained before enrollment and any study‐specific assessment. Recruitment procedures were designed to ensure equitable and impartial participant selection across the participating sites.

### 2.3 Eligibility Criteria

Participants were eligible if they were aged between 45 and 70 years, had a KOA diagnosis as defined by the American College of Rheumatology, had a Kellgren‐Lawrence index of II or III, and signed the informed consent form. Exclusion criteria included systemic inflammatory arthritis; recent oral steroid use within 3 months; recent knee treatment or corticosteroid injections within 6 months; previous knee surgery; neurological deficits; cardiopulmonary conditions that prevent exercise; and contraindications to KT, such as severe skin injuries, open wounds, allergies, and pregnancy [4, 19, 20]. We aim to ensure equal and impartial recruitment.

### 2.4 Randomization and Blinding

Participants were randomly assigned in a 1:1 ratio to receive KT with NCE or sham KT with NCE. Randomization was carried out using sequentially numbered, opaque, sealed envelopes prepared by an independent researcher who was not involved in recruitment or assessment. The allocation was revealed only after the baseline assessment. Baseline, post‐intervention, and follow‐up assessments are conducted by blinded outcome assessors, with different assessors assigned to each group. Intervention delivery and KT application are performed by separate therapists who are not involved in outcome assessments. The statistician will remain blinded to group allocation until the analysis is complete. Due to the nature of the intervention, participant and therapist blinding was not possible. The success of blinding will be evaluated after the study concludes.

### 2.5 Interventions

All intervention sessions are conducted by experienced, qualified physiotherapists who received training prior to the study and were provided with treatment manuals and checklists to ensure adherence. Regardless of group assignment, all participants receive usual care at the physiotherapy clinics, which includes education on joint protection, advice on activity modification, and general lifestyle recommendations.

Between baseline and the 8 weeks post‐intervention, participants assigned to the experimental group receive a supervised exercise program combining NCE and KT. Each week comprises three face‐to‐face sessions of approximately 60 min, totaling 24 sessions over the intervention period. Sessions begin with a 10‐min warm‐up (ergometer cycling, treadmill walking, or stepper), followed by NCE, including quadriceps and hamstring strengthening, lunging, pelvic tilting, step‐ups, squatting, weight shifting, mini‐trampoline activities, limping cross, cloth under foot, kettlebell swings, side‐lying jumping jacks, elastic band exercises, and gait training. Exercises progress through three levels, with each performed in 1–3 sets of 15 repetitions. Sessions conclude with a 10‐min cool‐down period consisting of alignment‐focused walking and stretching of the lower extremity muscles. Table 2 provides a clear and detailed description of the NCE protocol [15, 21].

**Table 2:** Neuromuscular control exercise (NCE) protocol.

Exercise component	Exercise/activity	Purpose and key features	Dose/progression
Warm‐up	Cycling, treadmill walking, or stepper	Low‐intensity whole‐body and lower‐limb warm‐up	10 min; modality selected according to participant tolerance
Muscle strengthening	Quadriceps and hamstring strengthening	Improve lower‐limb muscle strength and neuromuscular control	1–3 sets × 15 repetitions; progress through 3 predefined levels
Functional movement	Squatting	Controlled hip and knee flexion–extension with emphasis on lower‐limb alignment	1–3 sets × 15 repetitions; difficulty progress according to performance
Functional loading	Step‐ups	Improve controlled weight acceptance and lower‐limb loading	1–3 sets × 15 repetitions; progress according to functional capacity
Dynamic postural control	Multidirectional weight shifting	Improve controlled weight transfer and postural stability	1–3 sets × 15 repetitions; progress from basic to dynamic loading
Resistance control	Elastic‐band exercises	Improve resisted lower‐limb movement and neuromuscular control	1–3 sets × 15 repetitions; resistance progress as tolerated
Dynamic coordination	Mini‐trampoline activities, limping cross, cloth‐under‐foot exercise, kettlebell swings, and side‐lying jumping jacks	Enhance dynamic balance, coordination, lower‐limb control, and functional movement	1–3 sets × 15 repetitions; progress through 3 predefined levels
Gait training	Alignment‐focused gait training	Promote controlled gait mechanics and lower‐limb alignment	Protocolized; progress according to movement quality and tolerance
Cool‐down	Alignment‐focused walking and lower‐extremity stretching	Facilitate recovery and maintain lower‐limb flexibility	10 min

*Note:* The NCE program is being delivered three times weekly for 8 weeks (24 sessions), with each session lasting approximately 60 min.

KT is being administered three times weekly over 8 weeks. Before the application, the skin is being cleansed, and participants are being positioned side‐lying with the hip extended and the knee flexed at 60°. An I‐shaped tape is being applied from the rectus femoris origin, with a Y‐shaped tape applied proximal to the superior border of the patella. The tape base is being applied without tension, while the segment directed toward the superior patella is being stretched by approximately 40%. Therapists are receiving standardized training to achieve the target stretch using manufacturer‐recommended reference markers and pre‐measured tape lengths. The tape is being removed after 48 h, and skin sensitivity is being monitored at each reapplication [4, 22].

Participants assigned to the control group are performing the same NCE protocol with sham KT. They are attending three supervised sessions per week for 8 weeks (24 sessions in total), with the same warm‐up, progression model, and cool‐down procedures as the experimental group. Exercise modifications are made when necessary to accommodate individual tolerance while maintaining overall protocol consistency. Sham KT is applied at the same frequency as KT, without stretching and with the knee extended. Any protocol deviations are documented for reporting in the final results. Participant tolerance and adverse events are monitored throughout the intervention using individual monitoring sheets to ensure safety.

### 2.6 Outcome Measurements

Outcome measures included sociodemographic information such as gender, body mass index (BMI), duration and severity of symptoms, radiological findings, comorbidities assessed using the Charlson Comorbidity Index (CCI), history of falls in the past year, physical activity, and other factors relevant to KOA. Primary outcomes focus on pain and functional status, while secondary outcomes include lower‐limb‐specific muscle strength, knee ROM, and functional mobility. Questionnaires were initially developed in English and then translated and back‐translated into Bengali by a bilingual researcher to ensure accuracy and consistency. All outcomes are planned to be assessed at baseline, post‐intervention (8 weeks), and follow‐up (6 months) using questionnaires and clinical examinations, with measurements conducted by trained, blinded assessors.

#### 2.6.1 Primary Outcomes

##### 2.6.1.1 Visual Analog Scale (VAS)

Pain intensity is being assessed using a simple 10 cm VAS, a widely used tool for measuring pain. It features a 10 cm horizontal line, with 0 representing “*no pain*” and 10 indicating “*severe pain*.” The VAS has shown excellent reliability in measuring KOA pain, with an ICC of 0.97 [23].

##### 2.6.1.2 Pressure Pain Threshold (PPT)

PPT of the affected knee joint is being assessed using a handheld digital pressure algometer (FPK 20, Wagner Instruments, USA), a validated method for PPT assessment in KOA. Measurements are taken at the most tender site associated with KOA, recorded three times at 30‐s intervals, and averaged for analysis (ICC = 0.91) [24].

##### 2.6.1.3 Functional Status

Functional status is being assessed using the Western Ontario and McMaster Universities Osteoarthritis Index (WOMAC), a validated instrument for assessing KOA. It includes 24 items across pain (ICC = 0.86), stiffness (ICC = 0.68), and physical function (ICC = 0.89), with sub‐scores of pain (0–20), stiffness (0–8), and physical function (0–68), totaling 96 [25].

#### 2.6.2 Secondary Outcomes

##### 2.6.2.1 Muscle Strength

Lower‐limb muscle strength is being evaluated using a modified sphygmomanometer (Tycos model DS44, NY, USA) that measures pressure from 0 to 300 mmHg, given its feasibility and cost‐effectiveness. This method is a validated, reliable, and clinically acceptable tool for measuring strength. Muscle strength in knee flexion and extension is being evaluated, as these have shown good reliability in previous research (ICC = 0.80–0.99) [26].

##### 2.6.2.2 Range of Motion

Knee joint ROM is being assessed utilizing a standard goniometer, a valid tool in clinical research and physiotherapy practice. Knee flexion–extension ROM is being assessed, a measure that has demonstrated excellent reliability in previous research (ICC = 0.99) [27].

##### 2.6.2.3 Timed UP and Go Test (TUG)

The TUG test is being used to evaluate functional mobility. The TUG records the time taken to rise from an armchair, walk 3 meters, and return to a fully seated position. Time is being measured in seconds, and a duration exceeding 12 s indicates a fall risk. This is a valid and reliable testing procedure for mobility impairments and falls in KOA patients (ICC = 0.96) [28].

### 2.7 Sample Size Calculation

G*Power, version 3.1.9.7 (University of Kiel, Kiel, Germany), was used to determine the required sample size for this study. The calculation indicates that a total of 68 participants are required, with an effect size (ES) of 0.61 for the functional outcomes, an *α* level of 0.05, and a power of 0.80 [29]. Therefore, each group requires 34 participants. After accounting for a 15% dropout rate, the number of participants in each group is increased to 40, resulting in a final estimated total of 80 participants.

### 2.8 Statistical Analysis

All statistical analyses will be performed using SPSS (Statistical Package for the Social Sciences), version 25 (IBM Corp., Armonk, NY, USA). Descriptive statistics will be presented as frequencies, percentages, means, and standard deviations, as appropriate. For between‐group comparisons of baseline characteristics, independent‐samples *t* tests will be used for continuous variables, and Pearson's Chi‐square (*χ* ^2^) tests will be used for categorical variables. Changes in outcome measures across the three assessment time points will be evaluated using repeated‐measures analysis of variance, with group, time, and group × time interaction effects examined to assess differences in trajectories between the intervention groups. Where appropriate, post hoc comparisons will be performed with adjustment for multiple comparisons. ESs will be estimated using partial eta squared (ηp^2^) for the repeated‐measures analyses and Cohen's d for pairwise comparisons, with Cohen's d values of 0.20, 0.50, and 0.80 interpreted as small, moderate, and large effects, respectively. Between‐group mean differences will be reported with corresponding 95% confidence intervals (CIs) to enhance the precision and clinical interpretability of the estimates. All analyses will follow the intention‐to‐treat principle, including all randomized participants in the analysis according to their assigned groups. Missing data will be addressed using multiple imputation under a missing‐at‐random assumption. A two‐sided *p*‐value of < 0.05 will be considered statistically significant.

### 2.9 Data Management and Monitoring

Data will be managed with strict measures to ensure accuracy and confidentiality, with each participant assigned a unique ID for anonymization. All information will be double‐verified and manually entered twice to minimize errors, then stored securely on encrypted servers under the lead investigator's responsibility. Access will be restricted to authorized personnel who are bound by confidentiality agreements. Statistical analyses will use anonymized datasets, and findings will be reported only in aggregate. Trial monitoring is conducted by two independent professionals who are not involved in the trial; they oversee participant enrollment, monitor adverse events, verify adherence to the intervention protocol, and perform periodic data reviews. They organize regular meetings to evaluate safety, study progress, data integrity, and protocol compliance, thereby ensuring the overall quality and reliability of the research.

### 2.10 Safety Precautions and Adverse Effects Management

Although the likelihood of intervention‐related adverse effects is low, participants are being monitored throughout the intervention for any unexpected events. Before each KT application, the skin is inspected for irritation, wounds, dermatitis, or other contraindications, and participants are asked about any previous tape‐related reactions. Participants are instructed to report itching, burning, pain, excessive tightness, redness, swelling, blistering, or other adverse reactions. If a mild tape‐related reaction occurs, the tape is removed and re‐application is withheld until symptoms resolve; moderate or severe reactions result in discontinuation of taping and appropriate clinical referral. All adverse events are documented and promptly reported to the principal investigator. Any serious or unexpected adverse event is managed in accordance with the study's safety‐monitoring procedures and reported to the relevant specialists and the Ethical Review Board, as required. Any necessary protocol modifications are communicated to the Ethical Review Board through the established monitoring procedures.

### 2.11 Study Status

The study is being conducted from September 2025 and is expected to be completed by December 2026.

### 2.12 Dissemination

The study results will be shared after completion through a special event for researchers, physiotherapists, and healthcare professionals. The findings will also be published in reputable open‐access journals for broad dissemination. Additionally, meetings with healthcare experts will be held to discuss the results and their impact, to improve treatment for patients with KOA.

### 2.13 Patient and Public Involvement

Patients and/or the public were not involved in the design, conduct, reporting, or dissemination plans of this research.

## 3 Results

This study is a protocol for a RCT; therefore, no outcome data have been collected, and results are not yet available. It is anticipated that both KT combined with NCE and sham KT with NCE will improve primary outcomes, including pain intensity, PPT, and functional status. Secondary outcomes are expected to improve lower‐limb muscle strength, knee ROM, and functional mobility. Between‐group analyses will assess whether the addition of KT provides superior clinical and statistical benefits compared with sham KT with NCE at post‐intervention (8 weeks) and follow‐up (6 months). Data on treatment adherence, adverse events, and intervention feasibility will also be reported. Final results will be disseminated through peer‐reviewed journals and scientific conferences.

## 4 Discussion

This study is a multicenter RCT evaluating the combined effectiveness of KT and NCEs compared with sham KT with NCE in individuals with KOA. While previous studies have investigated KT and NCE independently, their synergistic effects have not been rigorously tested within a standardized protocol featuring long‐term follow‐up. The novelty of this trial lies in its evaluation of KT as an adjunct to a structured exercise program in Bangladesh, contributing new evidence from a South Asian context where such research is scarce. By determining whether integrating KT with NCE produces superior outcomes in pain reduction, functional improvement, and physical performance, this study aimed to establish a practical, evidence‐based approach to KOA rehabilitation in resource‐limited settings.

KOA is one of the most common musculoskeletal disorders worldwide, characterized by pain, stiffness, reduced mobility, and progressive functional decline [1]. Non‐pharmacological management remains the mainstay of treatment, with exercise therapy consistently recommended as the most effective first‐line approach [15]. Among exercise modalities, NCE has gained attention for its ability to improve joint alignment, enhance neuromuscular coordination, strengthen periarticular muscles, and decrease joint loading. Clinical evidence suggests that NCE can improve balance, proprioception, muscle strength, and patient‐reported outcomes, even in older adults with moderate‐to‐severe KOA [15, 16, 17, 18]. Simultaneously, KT has become a widely used adjunct in physiotherapy practice [4]. It is believed to improve local circulation, reduce pain and swelling, enhance proprioceptive feedback, and provide functional stability to the knee joint [10]. Several trials and systematic reviews have reported that KT, when used alone or in combination with conventional physiotherapy, can reduce pain and improve physical function in KOA, although some studies have produced conflicting results [4]. The inconsistent evidence highlights the need for a robust, well‐designed RCT to clarify the true contribution of KT when added to exercise therapy.

The present study, therefore, justifies its intervention design by integrating KT with NCE within a structured rehabilitation protocol. KT is anticipated to reduce pain and improve movement, enabling patients to perform NCE more effectively and consistently. Conversely, NCE targets neuromuscular and biomechanical deficits that contribute to functional limitations in KOA [4]. Together, these methods may offer a synergistic effect, boosting both immediate outcomes, such as pain relief and enhanced mobility, and long‐term benefits, including sustained independence and improved quality of life. The selection of outcomes—including pain intensity, PPT, functional status, muscle strength, ROM, and functional mobility—ensures a comprehensive assessment of patient progress. Furthermore, incorporating both subjective and objective measures, along with a 6‐month follow‐up, is likely to yield stronger evidence than prior studies with a short‐term or single‐outcome focus. Patients recruited from multiple centers across Bangladesh will enable a diverse sample, thus enhancing the external validity within the regional context.

The study findings could significantly benefit KOA management practice by endorsing a safe, cost‐effective, and easily implementable intervention that requires neither sophisticated equipment nor extensive financial investment. The strengths of this study include its multicenter design, assessor blinding, adequate sample size, standardized intervention protocol, and long‐term follow‐up. These methodological features enhance the internal validity and reliability of the results. However, some limitations should be considered. The study is being conducted in a Bangladeshi population, which may limit the generalizability of the findings to other populations and healthcare settings. Variations in exercise adherence and follow‐up participation may influence the study outcomes. Although adverse events are expected to be minimal, some participants may experience discomfort or delayed‐onset muscle soreness, which may affect their adherence to or engagement with the intervention. Furthermore, as both groups are receiving the same NCE program, the study is primarily designed to determine the additional effect of KT rather than the independent effects of KT or NCE alone. Future research should investigate the long‐term sustainability, cost‐effectiveness, and cross‐cultural applicability of KT combined with NCE or other exercise interventions, while incorporating advanced radiological imaging to assess structural changes across different stages of KOA.

## 5 Conclusion

This study will strengthen the evidence for non‐pharmacological management of KOA by assessing the combined effects of KT and NCE. By determining whether this integrated approach provides greater benefits than exercise alone, it aims to establish an effective, safe, and sustainable rehabilitation strategy. The findings are anticipated to inform clinical guidelines, enhance patient outcomes, and support the development of cost‐effective interventions to lessen the global impact of KOA.

## Author Contributions

**Md Zahid Hasan:** conceptualization, investigation, methodology, project administration, writing – original draft, writing – review and editing. **Kazi Md Azman Hossain:** conceptualization, investigation, methodology, project administration, writing – original draft, writing – review and editing. **Mohammad Anwar Hossain:** conceptualization, investigation, methodology, supervision, writing – review and editing. **Md Obaidul Haque:** data curation, supervision, funding acquisition, writing – review and editing. **Farjana Sharmin:** data curation, software, funding acquisition, writing – review and editing, visualization. **Farzana Sharmin:** writing – review and editing, funding acquisition, validation, resources, formal analysis. **Md Zahid Hossain:** conceptualization, investigation, methodology, funding acquisition, project administration, writing – original draft, writing – review and editing.

## Funding

The authors have nothing to report.

## Ethics Statement

All procedures adhere to the 2020 Declaration of Helsinki and the institution's established ethical guidelines. The research has obtained ethical approval from the Physiotherapy Rehabilitation and Research Ethics Committee of the Bangladesh Physiotherapy Association (ID: BPA‐IPRR/IRB/2024/12/1086) and is registered in the Clinical Trial Registry of India (ID: CTRI/2025/08/093317).

## Consent

Before enrolling in the study, written consent was obtained from participants and their guardians.

## Conflicts of Interest

The authors declare no conflicts of interest.

## Transparency Statement

The corresponding author (Md Zahid Hossain) affirms that this manuscript is an honest, accurate, and transparent account of the study being reported; that no important aspects of the study have been omitted; and that any discrepancies from the study as planned have been explained.

## Data Availability

The data that support the findings of this study are available from the corresponding author upon reasonable request.
